# Spatiotemporal trends and covariates of Lyme borreliosis incidence in Poland, 2010–2019

**DOI:** 10.1038/s41598-024-61349-z

**Published:** 2024-05-10

**Authors:** Joanna Kulisz, Selwyn Hoeks, Renata Kunc-Kozioł, Aneta Woźniak, Zbigniew Zając, Aafke M. Schipper, Alejandro Cabezas-Cruz, Mark A. J. Huijbregts

**Affiliations:** 1 Chair and Department of Biology and Parasitology, Medical University of Lublin, Radziwiłłowska St. 11, 20-080 Lublin, Poland; 2 Department of Environmental Science, Radboud Institute for Biological and Environmental Sciences, Radboud University, P.O. Box 9010, 6500 Nijmegen, GL The Netherlands; 3 Anses, UMR BIPAR, Laboratoire de Santé Animale, INRAE, Ecole Nationale Vétérinaire d’Alfort, 94700 Maisons-Alfort, France

**Keywords:** Infectious diseases, Ecological epidemiology, Ecological modelling, Environmental impact

## Abstract

Lyme borreliosis (LB) is the most commonly diagnosed tick-borne disease in the northern hemisphere. Since an efficient vaccine is not yet available, prevention of transmission is essential. This, in turn, requires a thorough comprehension of the spatiotemporal dynamics of LB transmission as well as underlying drivers. This study aims to identify spatiotemporal trends and unravel environmental and socio-economic covariates of LB incidence in Poland, using consistent monitoring data from 2010 through 2019 obtained for 320 (aggregated) districts. Using yearly LB incidence values, we identified an overall increase in LB incidence from 2010 to 2019. Additionally, we observed a large variation of LB incidences between the Polish districts, with the highest risks of LB in the eastern districts. We applied spatiotemporal Bayesian models in an all-subsets modeling framework to evaluate potential associations between LB incidence and various potentially relevant environmental and socio-economic variables, including climatic conditions as well as characteristics of the vegetation and the density of tick host species. The best-supported spatiotemporal model identified positive relationships between LB incidence and forest cover, the share of parks and green areas, minimum monthly temperature, mean monthly precipitation, and gross primary productivity. A negative relationship was found with human population density. The findings of our study indicate that LB incidence in Poland might increase as a result of ongoing climate change, notably increases in minimum monthly temperature. Our results may aid in the development of targeted prevention strategies.

## Introduction

Lyme borreliosis (LB) is the most commonly diagnosed tick-borne disease in the northern hemisphere^1–4^. It is estimated that over 1,000,000 people worldwide are affected by LB each year, with approximately 25% of cases occurring in Europe. The highest incidence rates among European countries are recorded in Belgium, Finland, the Netherlands, and Switzerland (> 100/100,000 inhabitants per year), and the lowest in Belarus, Croatia, Denmark, France, Ireland, Portugal, and the United Kingdom (except Scotland) (< 20/100,000 inhabitants per year)^5,6^. In Poland, the incidence of LB is at a level similar to that reported from neighboring countries, i.e., Czech Republic and Germany (20–40 cases per 100,000 inhabitants per year). The yearly economic costs associated with diagnosing and treating LB are substantial, surpassing $20 million in the Netherlands, over $40 million in Germany, and ranging from $800 million to over $3 billion in the United States^7–10^.

The agents of LB are spirochetes of the *Borreliella* genus, primarily *B. burgdorferi* in North America and *B. afzelii*, *B. garinii, B. bavariensis and B. spielmanii* in Europe. Ticks of the *Ixodes* genus, including *I. scapularis* and *I. pacificus* in North America and *I. persulcatus* and *I. ricinus* in Eurasia, are the primary vectors for *Borreliella* spirochetes^1,11,12^. *Ixodes ricinus* is widely distributed across Europe, has a non-specific host range, and plays a crucial role in the enzootic circulation of *Borreliella* spp. spirochetes^13–15^. The broad feeding capability of *I. ricinus*, enabling it to feed on over 300 vertebrate species from diverse taxonomic groups occurring in natural, urban, and suburban environments, greatly enhances the circulation and transmission of LB across the European continent^16–18^.

People spending time in ticks-populated habitats are at the highest risk of infection. Since an effective vaccine is currently not available, prevention of transmission is essential^19–21^. This, in turn, requires a good understanding of the spatio-temporal dynamics of LB transmission as well as the underlying factors. It is well-known that climatic factors, including temperature and precipitation, affect tick distribution and the prevalence of tick-borne pathogens^12,22^. The presence of potential tick hosts and the structure of their communities also influence ticks’ behavior, pathogen prevalence, and the risk of pathogen transmission^16,23^. Tick distribution and pathogen prevalence are further related to vegetation. Especially deciduous forests provide a suitable habitat for *I. ricinus*, due to favorable humidity conditions and the support of its hosts. Still, also urban green areas are inhabited by pathogen-infected ticks and pose a risk for disease transmission to humans^16,21,24^. The multitude of underlying environmental factors makes it challenging to understand their relative and combined contribution to LB incidence.

Statistical modeling based on reliable and consistent data is a useful tool to identify and unravel the different factors, which in turn can help to predict disease incidence, identify high-risk areas, and develop LB-focused educational and prophylaxis programs^21,25^. Unfortunately, large-scale modeling can be challenging due to differences in data collection systems, storage, availability, and legal requirements for reporting LB incidence within and between countries^26^. For instance, the European Centre for Disease Control and Prevention (ECDC) collects and presents reported data only on neuroborreliosis, which for Poland in 2019 constituted about 1.5% of all LB cases. In Poland, all diagnosed forms of LB are reported to the National Institute of Public Health – National Institute of Hygiene (NIPH-NIH)^27,28^.

In this study, we aimed to identify spatiotemporal trends and unravel covariates of LB incidence in Poland by using a unique and consistent dataset with yearly incidences collected from 380 Polish districts (organized into 320 territorial units) from 2010 through 2019. The dataset enabled us to systematically explore how the LB incidence is related to various relevant environmental and socio-economic factors, including climatic conditions, vegetation and tick host community characteristics, and human population density, covering the territory of the country up to the highest possible spatial resolution (district level). To provide a comprehensive understanding of potential associations between LB incidence and environmental or socio-economic variables, we utilized conditional autoregressive Bayesian models, accounting for spatiotemporal autoregressive processes^29,30^.

## Results

### Lyme borreliosis incidence in Poland

**Figure 1:**
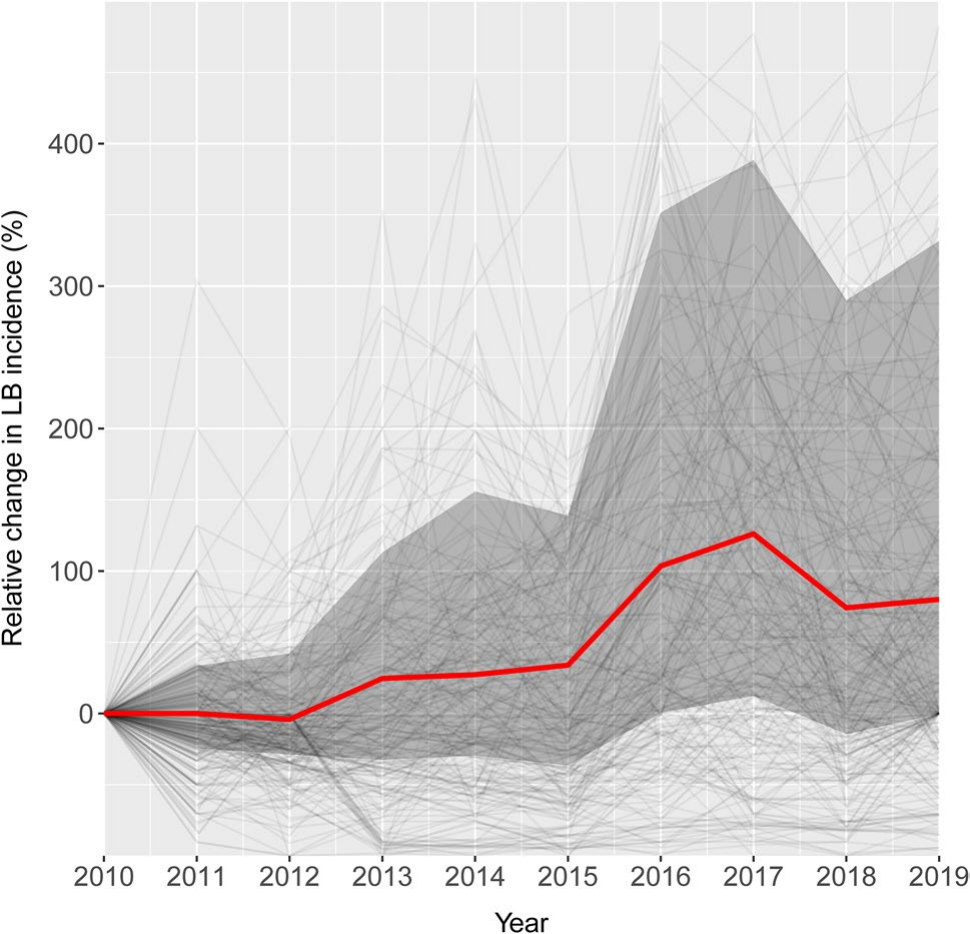
Relative change in Lyme borreliosis incidence per 100,000 inhabitants from 2010 to 2019. The red line depicts the median percentage change across all 320 (aggregated) districts. The grey-shaded area shows the interquartile range (25th and 75th percentiles) of all data. The light-grey lines show the individual district trends. This figure was generated with the use of R (version 4.3.2) using ggplot2 (version 3.4.4).

**Figure 2:**
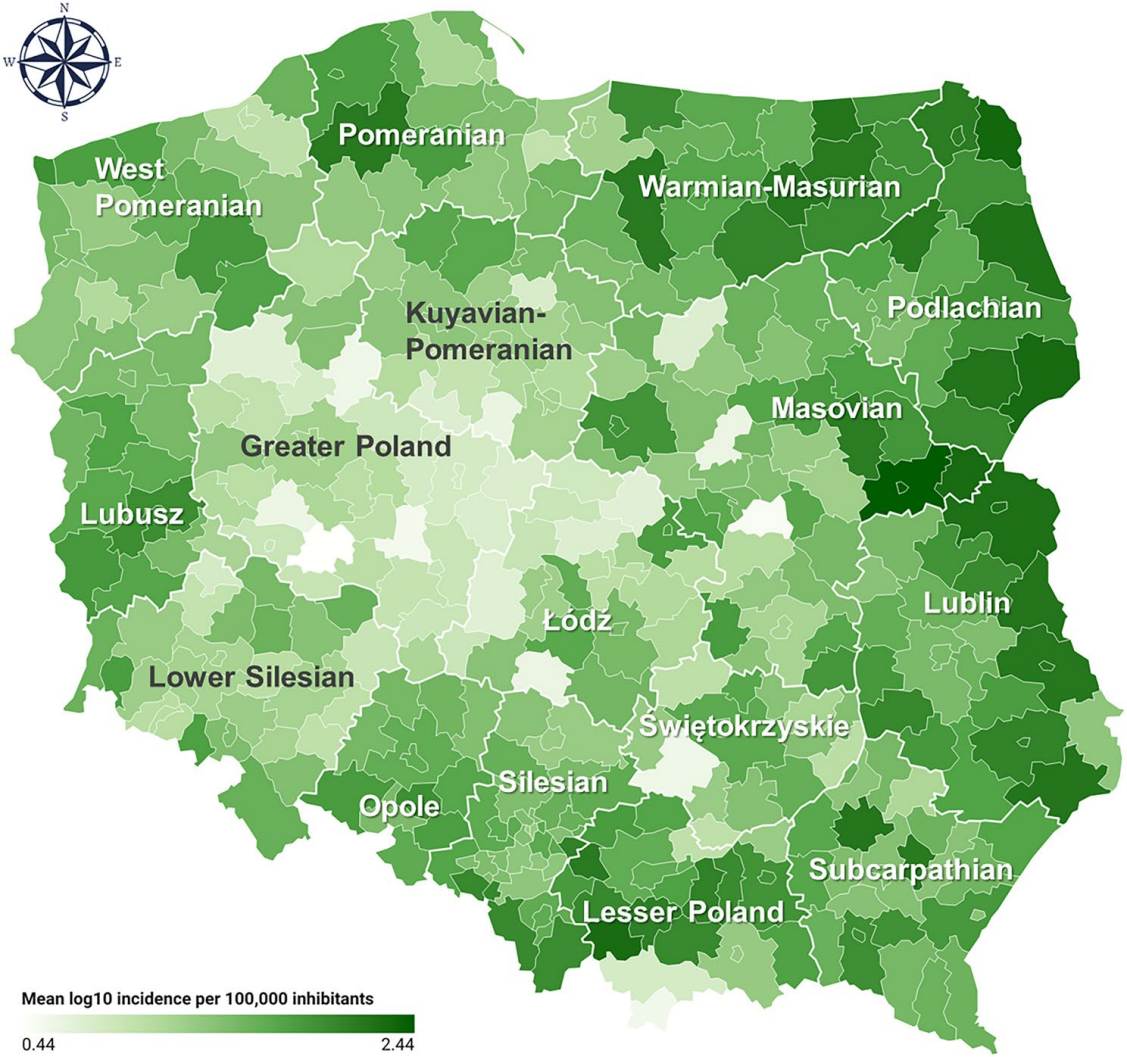
Mean Lyme borreliosis incidence per 100,000 inhabitants from 2010 to 2019 (log_10_ transformed) for all 320 aggregated districts. This map was generated based on the compiled LB incidence data using an online tool (https://www.datawrapper.de/).

Our analysis of the trends in LB incidence indicated a clear overall increase of LB cases in Poland during the studied period, yet with considerable variation in the trends between the districts (Fig. 1). Based on mean incidence data over 2010–2019, we identified high-risk LB transmission regions in the eastern, north-eastern and southern districts, with mean values per 100,000 inhabitants reaching up to 276.2 in district located in Masovian Voivodeship, 213.3 in Podlachian, 192.3 in Lesser Poland, 176.5 in Lublin and 156.8 in Warmian-Masurian Voivodeship (Fig. 2; Supplementary Fig. S1, Supplementary Table S1).

### Modelling results

**Figure 3:**
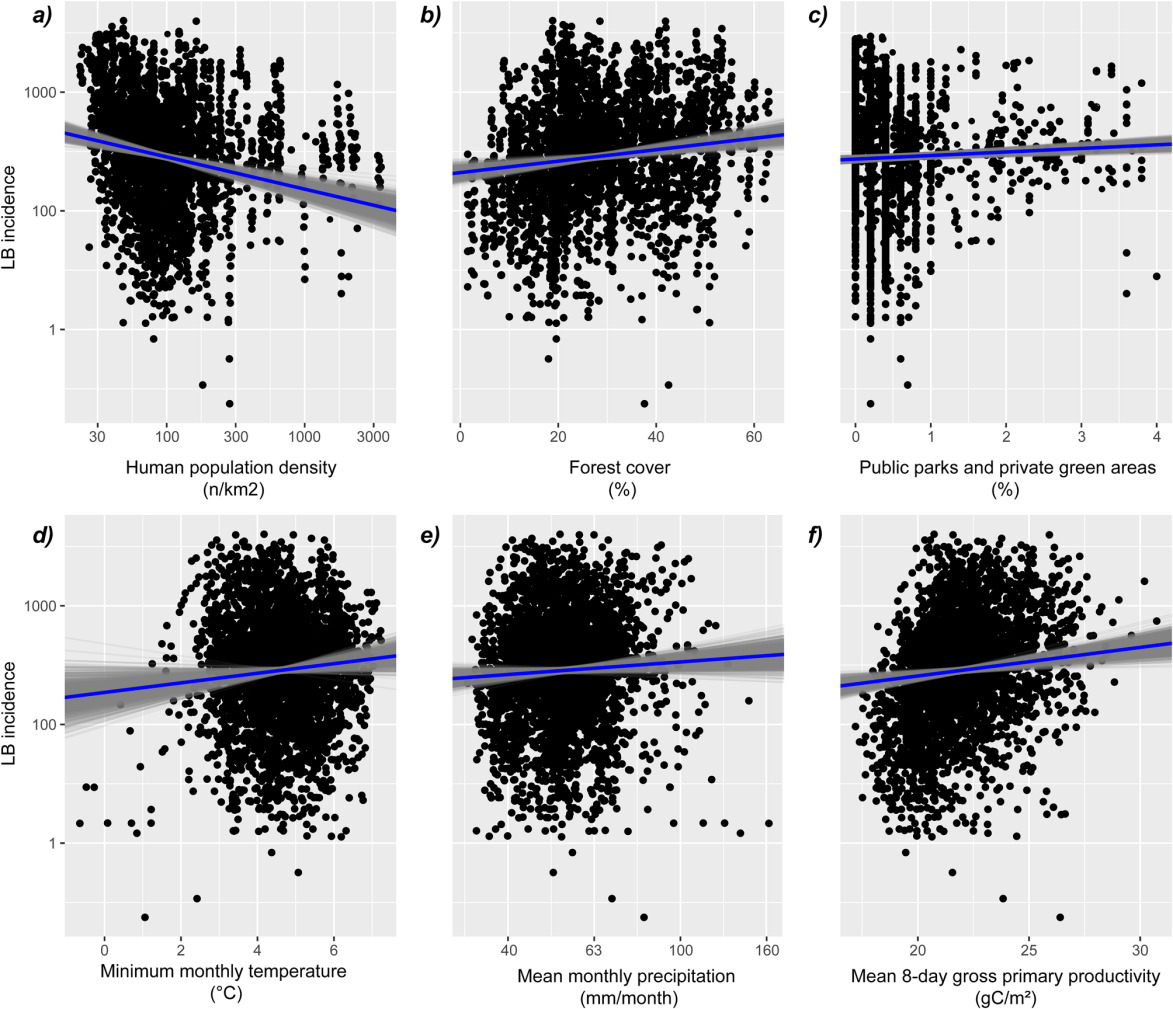
Partial response plots based on the posterior distributions for the fixed variables retained in the most parsimonious model. Blue lines depict the mean posterior response, and the grey lines show the individual Markov chain Monte Carlo samples (*n* = 1000). Black dots represent the raw data points. This figure was generated with the use of R (version 4.3.2) using ggplot2 (version 3.4.4).

The most parsimonious spatiotemporal Bayesian model revealed positive relationships between the incidence of LB and the following factors: annual mean monthly precipitation (mm/month), the share of parks, lawns, and green areas in housing estate areas (%), annual minimum monthly temperature (^o^C), yearly mean 8-day gross primary productivity (gC/m^2^) and the percentage of area covered by forest (Fig. 3; Supplementary Table S2). In contrast, we found a negative relationship between LB incidence and human population density (individuals/km^2^) (Fig. 3). The posterior distributions of the factors retained in the final model showed credible intervals (95% CI) that did not include zero (Fig. 3), except for the mean monthly precipitation (mean of 0.27 and 95% CI of − 0.03 to 0.56; Supplementary Table S2). The most parsimonious model had a Deviance Information Criterion (DIC) value that was approximately five points lower compared to the second lowest DIC value found during the model selection.

The spatial and temporal autoregressive parameters estimated in the final model had a mean of 0.74 (0.64 to 0.84 95% CI) and a mean of 0.88 (0.85 to 0.91 95% CI), respectively, confirming strong spatiotemporal autocorrelation in the data (Supplementary Table S2). Applying the Moran’s I test on the residuals of our final model showed that the spatial autocorrelation in the residuals was removed (*p* > 0.05).

The time trend of predicted median LB incidence across all districts showed a strong resemblance to the yearly median observed values (Supplementary Fig. S2), indicating that the model captures the temporal trend well. Using the symmetric mean absolute percentage error (sMAPE), we evaluated the predictive accuracy of our model on the district level. The calculated sMAPE-values ranged between 1.1% and 31.2%, with an average sMAPE-value of 5.63% across the districts.

## Discussion

Tick-borne diseases, including LB, are among the most frequently diagnosed human infectious diseases in Europe^5,27^. Hence, a solid understanding of the factors influencing their incidence is extremely important. Our results indicate that LB transmission in Poland is especially prevalent in the eastern regions (Fig. 2, Supplementary Fig. S1, Supplementary Table S1). Mean LB incidence in Poland during the studied period ranged from 2.7 up to 276 cases per 100,000 inhabitants across the districts (Supplementary Table S1), which is higher than values reported from countries from the region^5,27^. Moreover, our spatiotemporal analysis revealed a substantial overall increase in LB incidence in Poland, with a median increase of ca. 80% from 2010 to 2019 (Fig. 1).

Based on consistent, long-term data covering the whole country up to the highest possible spatial resolution (district level), our research revealed that LB incidence in Poland is related to various environmental and socio-economic factors. Specifically, our analysis revealed a positive relationship between LB incidence and forest cover in Poland, which is in line with findings reported previously in the United States^31^. While ticks tend to hide in forest vegetation and litter, where they can reabsorb water and conserve energy during periods of non-feeding, forests also support a diverse range of ticks’ potential hosts, including mammals and birds^32–35^. Increased forest cover in a region, including forests accessible to the public, raises the risk of ticks’ interactions with humans, leading to pathogen transmission^36^. Next to forests, other green areas, such as parks, gardens, and lawns, may also provide suitable microhabitats for ticks and their hosts and are linked to LB transmission, as revealed by our analysis and in line with previous studies^37,38^. People living near forests and other types of green areas or engaging in recreational or professional activities, such as foresters, outdoor workers, gardeners, and athletes, face an elevated risk of tick bites and *Borreliella* spirochetes transmission^19,20^.

We further found that LB incidence increases with increasing minimum monthly temperature (Fig. 3). The importance of climatic conditions is in line with the findings of previous studies showing that ticks activity depends on local as well as large-scale climatic conditions, including temperature and humidity^33,35,39^. As the *I. ricinus* ticks overwinter buried in the litter, severe winter conditions can affect their survival and following spring activity^40^. Low temperatures may also negatively affect rodents, which are primary vectors for juvenile stadia, and deer, which are one of the hosts of *I. ricinus* adults^41^. Therefore, increases in minimum temperatures caused by climate change may lead to an increased risk of LB transmission. In this context, it is worth noting that particular microorganisms may also promote ticks’ winter survival, as it was reported that *B. burgdorferi*-infected females of *I. scapularis* had increased overwintering ability in comparison to uninfected ticks ^42^.

Our model revealed a positive relationship between LB incidence and annual average 8-day gross primary productivity (GPP) (gC/m^2^). As GPP is a measure of ecosystem productivity in terms of energy and/or biomass production by primary producers, the positive relationship indicates that increases in ecosystem productivity may support local tick populations by creating suitable microhabitats (including critters), and by mitigating adverse environmental conditions^43–45^. A higher ecosystem productivity may also benefit herbivores and predators, which are common tick hosts, thus contributing to an increased transmission to humans^46^. However, the density of mammals was not retained as a covariate in our best-supported model.

Moreover, our results confirmed a positive relationship between LB incidence and mean monthly precipitation. This is consistent with studies showing that ticks, including *I. ricinus,* are associated with microhabitats characterized by high humidity^47^. A suitable range of humidity promotes ticks’ host-seeking behavior and development, including embryogenesis, hatching, and molting^40^. Furthermore, humid conditions in the ticks’ microhabitats also affect the biology and phenology of their hosts, which may impact ticks’ success in both host-seeking and feeding^33,48^. It is worth underlining that changes in humidity may have long-term and delayed consequences for the risk of pathogen transmission since the life cycle of *I. ricinus* could last up to several years ^40^. As humidity is linked to precipitation, changes in the magnitude and frequency of precipitation events due to climate change may alter the risk of LB transmission^32,33,39^.

Finally, we found a negative relationship between the incidence of LB and human population density (see Fig. 3). Highly urbanized areas, including large cities and agglomerations, have the highest human population density in Poland^49^. These areas are drastically influenced by human activities, likely reducing the availability of habitats of ticks and their hosts, resulting in decreased tick abundance and reduced risk of human-tick contact. On the other hand, increased human presence in developing suburbs and rural areas, as in the construction of houses and settlements in tick occurrence areas, can elevate the risk of pathogen transmission to humans and pets^18^. Adverse characteristics of urban habitats may be mitigated by improved microclimatic conditions, as cities located in temperate zones may be more suitable for ticks (even if their local populations are relatively small), due to slightly higher mean annual temperatures compared to surrounding areas^50^.

Finally, the spatial autocorrelation presented by the posterior distribution of the spatiotemporal random effects can be attributed to differences between districts not captured by the covariates included in our model (Supplementary Table S2). For example, the territory of Poland is characterized by varied topography, from lowlands in the north to mountains in the south of the country, including areas of lakelands and highlands, which may locally impact microclimatic conditions influencing both ticks and their hosts. Additional factors that may affect LB incidence include land cover types, specifically agriculturally used and fallow lands, as well as ecotones – transition zones between diverse types of ecosystems^37,51,52^. We also note that we did not consider the density of tick populations and the proportion of *Borreliella*-infected specimens. Although available data indicate that *I. ricinus*, the main vector of LB in Poland, can be found across the country, its occurrence is characterized by a patchy distribution^53,54^. Furthermore, *Borreliella* spp. prevalence in ticks varies across Poland^55,56^. However, the incorporation of these factors into country-wide analyses is hampered by differences in the methodologies applied for tick collection and pathogen detection^55^. Moreover, human behavior may also affect the risk of LB transmission, for example through encroachment into ticks’ habitats during recreation and traveling, as well as the ‘urbanization’ of tick species together with their hosts^18^. Follow-up research is needed to get a better understanding of the influence of these factors on LB incidence.

## Conclusions

Based on detailed epidemiological data gathered on the level of districts in Poland for the period 2010–2019, we were able to analyze spatio-temporal trends in the incidence of LB in Poland and link it to vegetation characteristics, climate factors, and socio-economic variables. The overall increase in LB incidence and potential future increases due to climate change justify increased attention in national health policy, for example via pathogen screening programs covering people in occupations associated with a high risk of LB transmission. Educating citizens about the disease, its vector, transmission routes, and preventive measures could also be a key component of the national health policy.

## Methods

### Lyme borreliosis incidence data collection and preparation

We obtained data on yearly Lyme borreliosis (LB) cases for 380 districts (in Polish: powiat) between 2010 and 2019 from each of the 16 Voivodeship Sanitary Stations in Poland, upon request. We excluded the years 2020–2022 from the analysis to avoid bias caused by the SARS-CoV-2 pandemic, as suggested by previously published papers^57^. The dataset encompassed a total of 3,140 yearly reported LB cases. In some cases, a single Sanitary Station covered multiple administrative districts and reported accumulated epidemiological data. As a result, our analysis is based on 320 individual or aggregated districts. To calculate LB incidences (cases per 100,000 inhabitants), we divided the number of cases by the number of inhabitants per district or aggregated district^58^ and we log-transformed the result to reduce the positive skew in the data distribution, as follows:

1 $$LB = \log_{10} \left( { \frac{{N_{LB} + 1}}{N} *100,000} \right)$$

where ${N}_{LB}$ and $N$ are the total number of LB cases and the total number of inhabitants for a specific year and district, respectively.

Subsequently, we calculated for each (aggregated) district the change in LB incidence relative to the first year using the following equation:

2 $$Change_{2010 - year} = \left( {\frac{{LB_{year} - LB_{2010} }}{{LB_{2010} }}} \right)*100\%$$

### Covariate data collection and processing

We compiled data on potentially relevant environmental and socio-economic factors associated with the incidence of LB for each year and district. We identified relevant covariates based on the literature (for details see Supplementary Table S3). We collected information on forest cover, the percentage of forest area dominated by specific tree species, the surface area of green spaces (including parks, lawns, and residential areas) as well as the total population of wild mammal species in each district from the Forest Data Bank^59^ and Local Data Bank^58^ (Supplementary Table S3). Regarding the forest cover, we aggregated the reported percentages of different tree species into two groups: deciduous or coniferous. For each district, the sum of the deciduous and coniferous groups equals 100%. These values were then multiplied by the fraction of the district area covered by forests to derive the relative area covered by deciduous or coniferous trees on a district level. Since data on tree dominance was available for 2012 to 2019, we assumed that the values of 2012 were representative of 2010 and 2011. As these data were available only from 2016 to 2021, we extrapolated linearly to estimate values for the missing years.

For obtaining climatic covariate data, we utilized various sources (TerraClimate, MODIS, LANDSAT 7, and ERA5) and employed the rgee R package in the Google Earth Engine^60–63^. We obtained year-specific data for climate variables from 2010 to 2019 (Supplementary Table S3). Specifically, we extracted the daily mean temperatures and calculated the growing degree days (GGD) based on the daily mean temperature values exceeding 5 ℃. We also extracted the yearly mean 8-day gross primary productivity (GPP) values (gC/m^2^) for each district. Furthermore, we gathered data on the yearly average monthly precipitation (mm/month) and the yearly number of days with snow cover (Supplementary Table S3). Since the climatic covariate data was in raster format, we computed average values per district for each year using district polygons from Humanitarian Data Exchange v1.72.0 PY3^64^.

Lastly, we compiled socio-economic covariate data from the Local Data Bank^58^, including the number of nurses and medical doctors per 100,000 inhabitants, as well as the total population count for each district (Supplementary Table S3). To calculate population density (n/km^2^) for each year, we divided the number of inhabitants by the surface area of the district polygon. The polygon area was calculated using the st_area() function included in the sf R package^65^.

### Model fitting

Before fitting models, we log_10_-transformed several variables because of their skewed distribution (see Supplementary Table S3; Supplementary Fig. S3; S4). Additionally, we assessed potential multicollinearity by calculating the variance inflation factor (VIF) for each covariate. We removed variables with a VIF greater than 3 to mitigate multicollinearity concerns. As a result, we excluded the maximum monthly temperature from the model selection procedure. Next, we assessed if the remaining covariates were able to capture the spatial and temporal autocorrelation in the LB incidence data. To that end, we specified a naive global regression model relating LB incidence (transformed via Eq. (1) to all remaining covariates (see Supplementary Table S3), ignoring any potential autocorrelation structure, and applying the Moran’s I statistic^66^ on the residuals of our model for each year in the dataset. The Moran’s I test shows strong residual spatial autocorrelation for the individual years in our data set (*p* < 0.05), with values ranging from 0.15 to 0.21.

To account for the spatiotemporal autocorrelation, we continued our analysis with a conditional autoregressive (CAR) Bayesian modeling framework relying on Markov chain Monte Carlo (MCMC) simulations^30^. Because the data compiled on LB incidence and potential covariates is partitioned into a set over areal units (districts) with multiple consecutive annual observations (from 2010 to 2019), we selected a Bayesian hierarchical model with first-order autoregressive processes^29^. This model includes random effects to account for any residual spatiotemporal autocorrelation presented by the data after considering the effects of the initial main covariates. We fitted the model using the CARBayesST R package^67^. We specifically used the ST.CARar() function, which incorporates the model suggested by Rushworth et al.^29^. The spatial association between the districts was described using functions from the spdep R package^68^, which generated a neighborhood matrix that indicates whether a pair of district polygons share a border, relying on the district polygons from Humanitarian Data Exchange v1.72.0 PY3^65^.

Following an all-subsets modeling approach, we fitted models with all possible combinations of covariates and selected the model with the lowest Deviance information criterion (DIC) as the best-supported model. The DIC is tailored to Bayesian model selection, where the posterior distributions have been generated by MCMC iterations^69^. Similar to the widely used Akaike’s information criterion (AIC), the DIC can select the model based on both the goodness of fit as well as the effective number of parameters^69^. In the model selection process, we excluded candidate models that included both the percentage of district area with forest cover and the forest cover dominated by either deciduous or coniferous tree species as covariates. We did this because the percentage of district area with forest cover already incorporates the combined effect of forest cover dominated by deciduous and coniferous tree species.

For the model selection procedure, we ran the ST.CARar() function with 220,000 MCMC samples. From these samples, 20,000 were removed to account for the burn-in process and the leftover samples were thinned by 10 to remove most of the autocorrelation. Iterating through all possible combinations of covariates, the best model according to the obtained DIC values was refitted. The global model containing all factors was fitted using three separate MCMC chains to quantify the between-to-within chain variation in the MCMC samples using the Gelman–Rubin diagnostic^70^ and detect if the longer MCMC chains would potentially achieve a scale reduction. The final model was run with 1,100,000 MCMC samples, with a 1,000,000 burn-in period and a thinning of 100, resulting in 1000 samples for model inference. The MCMC sample size used in the model selection procedure was smaller compared to the final fit (factor of 5) for computational reasons. The MCMC sample size used for model selection (sample = 220,000; burn-in = 20,000; thinning = 10) was assessed by computing the Gelman–Rubin diagnostic^70^ for 3 individual chains for the global model (containing all variables). This resulted in a Gelman–Rubin diagnostic of 1.01, indicating that the selected MCMC sample size was sufficient (< 1.1). The convergence diagnostics for MCMC runs are presented as trace plots in Supplementary Fig. S4. The MCMC sample size used to refit the final model (sample = 1,100,000; burn-in = 100,000; thinning = 100) also showed to be sufficiently large according to the Gelman–Rubin diagnostic (< 1.1).

Using the symmetric mean absolute percentage error (sMAPE), we evaluated the predictive accuracy of our model by comparing the yearly predicted time trend to the observed time trend for each district individually. The entire analysis was performed in R version 4.3.2, using the ‘base’, ‘sf, ‘gstat’, ‘maptools’, ‘ggplot, ‘fst, ‘spdep’, and ‘CARBayesST’ packages^67,68^.

### Supplementary Information

Supplementary Information.

## Data Availability

All data compiled in this study has been published in the manuscript or Supplementary Information files.
