# Supplementary material for: Spatiotemporal trends and covariates of Lyme borreliosis incidence in Poland, 2010–2019
Source: Sci Rep. 2024 May 10;14:10768. doi: 10.1038/s41598-024-61349-z (PMC11087522; doi:10.1038/s41598-024-61349-z)

Supplementary Information

Spatiotemporal trends and covariates of Lyme borreliosis incidence in Poland, 2010-2019

Joanna Kulisz[1]*, Selwyn Hoeks[2], Renata Kunc-Kozioł[1], Aneta Woźniak[1], Zbigniew Zając[1], Aafke M. Schipper[2], Alejandro Cabezas-Cruz[3], Mark A.J. Huijbregts[2]

[1]Chair and Department of Biology and Parasitology, Medical University of Lublin, Radziwiłłowska St. 11, 20-080, Lublin, Poland

[2]Department of Environmental Science, Radboud Institute for Biological and Environmental Sciences, Radboud University, P.O. Box 9010, 6500, GL Nijmegen, the Netherlands

[3]Anses, INRAE, Ecole Nationale Vétérinaire d'Alfort, UMR BIPAR, Laboratoire de Santé Animale, Maisons-Alfort, F-94700, France

*Author to whom correspondence should be addressed

Supplementary Table S1. Mean incidence of Lyme borreliosis in Poland 2010-2019

Voivodeship	Territorial identifier	Districts	Mean incidence (per 100,000 inhabitants)	Mean incidence (per 100,000 inhabitants) log10 transformed
Lower Silesia	0201	Bolesławiecki	20.4	1.31
	0202	Dzierżoniowski	16.6	1.22
	0203	Głogowski	6.9	0.84
	0204	Górowski	33.4	1.52
	0205	Jaworski	16.3	1.21
	0206; 0261	Karkonoski aggregated	12.3	1.09
	0207	Kamiennogórski	63.0	1.80
	0208	Kłodzki	43.1	1.63
	0209; 0262	Legnicki aggregated	16.3	1.21
	0210	Lubański	67.8	1.83
	0211	Lubiński	30.8	1.49
	0212	Lwówecki	18.7	1.27
	0213	Milicki	41.6	1.62
	0214	Oleśnicki	8.9	0.95
	0215	Oławski	10.8	1.03
	0216	Polkowicki	10.1	1.00
	0217	Strzeliński	10.8	1.03
	0218	Średzki	11.8	1.07
	0219	Świdnicki	19.6	1.29
	0220	Trzebnicki	26.4	1.42
	0221; 0265	Wałbrzyski aggregated	29.9	1.48
	0222	Wołowski	40.5	1.61

	0223; 0264	Wrocławski aggregated	16.3	1.21
	0224	Ząbkowicki	29.4	1.47
	0225	Zgorzelecki	40.4	1.61
	0226	Złotoryjski	16.9	1.23
Kuyavian-Pomeranian	0401	Aleksandrowski	13.7	1.14
	0402	Brodnicki	27.4	1.44
	0403; 0461	Bydgoski aggregated	23.7	1.37
	0404	Chełmiński	19.2	1.28
	0405	Golubsko-Dobrzyński	16.9	1.23
	0406; 0462	Grudziądzki aggregated	32.4	1.51
	0407	Inowrocławski	9.6	0.98
	0408	Lipnowski	13.5	1.13
	0409	Mogileński	5.8	0.76
	0410	Nakielski	23.9	1.38
	0411	Radziejowski	4.4	0.64
	0412	Rypiński	20.8	1.32
	0413	Sępoleński	19.2	1.28
	0414	Świecki	52.4	1.72
	0415; 0463	Toruński aggregated	19.1	1.28
	0416	Tucholski	52.7	1.72
	0417	Wąbrzeski	6.4	0.81
	0418; 0464	Włocławski aggregated	16.7	1.22
	0419	Żniński	13.0	1.11
Lublin	0601; 0661	Bialski aggregated	176.5	2.25
	0602	Biłgorajski	59.5	1.77
	0603; 0662	Chełmski aggregated	150.4	2.18
	0604	Hrubieszowski	23.7	1.37
	0605	Janowski	75.7	1.88
	0606	Krasnostawski	68.0	1.83
	0607	Kraśnicki	106.2	2.03
	0608	Lubartowski	40.7	1.61
	0609; 0663	Lubelski aggregated	32.2	1.51
	0610	Łęczyński	29.9	1.48
	0611	Łukowski	34.0	1.53
	0612	Opolski	75.0	1.88
	0613	Parczewski	100.8	2.00
	0614	Puławski	44.7	1.65
	0615	Radzyński	83.3	1.92
	0616	Rycki	25.8	1.41
	0617	Świdnicki	40.5	1.61
	0618	Tomaszowski	156.4	2.19
	0619	Włodawski	158.9	2.20
	0620; 0664	Zamojski aggregated	118.3	2.07

Lubusz	0801; 0861	Gorzowski aggregated	30.7	1.49
	0802	Krosnieński	52.4	1.72
	0803	Międzyrzecki	33.8	1.53
	0804	Nowosolski	34.1	1.53
	0805	Słubicki	36.8	1.57
	0806	Strzelecko-drezdenecki	33.6	1.53
	0807	Sulęciński	59.4	1.77
	0808	Świebodziński	49.3	1.69
	0809; 0862	Zielonogórski aggregated	92.8	1.97
	0810	Żagański	55.6	1.74
	0811	Żarski	73.7	1.87
	0812	Wschowski	15.7	1.20
Łódź	1001	Bełchatowski	52.2	1.72
	1002	Kutnowski	7.6	0.88
	1003	Łaski	18.2	1.26
	1004	Łęczycki	8.8	0.94
	1005	Łowicki	5.3	0.72
	1006; 1061	Łódzki aggregated	11.8	1.07
	1007	Opoczyński	14.8	1.17
	1008	Pabianicki	46.1	1.66
	1009	Pajęczański	4.2	0.62
	1010; 1062	Piotrkowski aggregated	33.6	1.53
	1011	Poddębicki	9.0	0.96
	1012	Radomszczański	35.8	1.55
	1013	Rawski	12.7	1.10
	1014	Sieradzki	5.4	0.73
	1015; 1063	Skierniewicki aggregated	68.5	1.84
	1016	Tomaszowski	13.6	1.13
	1017	Wieluński	26.0	1.42
	1018	Wieruszowski	8.5	0.93
	1019	Zduńskowolski	19.6	1.29
	1020	Zgierski	5.8	0.77
	1021	Brzeziński	16.2	1.21
Lesser Poland	1201	Bocheński	119.6	2.08
	1202	Brzeski	99.6	2.00
	1203	Chrzanowski	138.4	2.14
	1204	Dąbrowski	19.7	1.29
	1205	Gorlicki	61.4	1.79
	1206; 1261	Krakowski aggregated	46.7	1.67
	1207	Limanowski	104.0	2.02
	1208	Miechowski	57.4	1.76
	1209	Myślenicki	126.7	2.10
	1210; 1262	Nowosądecki aggregated	21.2	1.33

	1211	Nowotarski	6.8	0.83
	1212	Olkuski	70.6	1.85
	1213	Oświęcimski	71.2	1.85
	1214	Proszowicki	10.6	1.02
	1215	Suski	192.3	2.28
	1216; 1263	Tarnowski aggregated	82.0	1.91
	1217	Tatrzański	3.7	0.57
	1218	Wadowicki	104.3	2.02
	1219	Wielicki	61.7	1.79
Masovian	1401	Białobrzeski	22.4	1.35
	1402	Ciechanowski	22.2	1.35
	1403	Garwoliński	34.0	1.53
	1404	Gostyniński	27.6	1.44
	1405	Grodziski	49.3	1.69
	1406	Grójecki	13.1	1.12
	1407	Kozienicki	31.0	1.49
	1408	Legionowski	35.7	1.55
	1409	Lipski	76.9	1.89
	1410	Łosicki	195.6	2.29
	1411	Makowski	26.9	1.43
	1412	Miński	18.4	1.26
	1413	Mławski	5.8	0.76
	1414	Nowodworski	4.2	0.63
	1415; 1461	Ostrołęcki aggregated	41.6	1.62
	1416	Ostrowski	63.5	1.80
	1417	Otwocki	52.7	1.72
	1418	Piaseczyński	3.2	0.51
	1419; 1462	Płocki aggregated	82.3	1.92
	1420	Płoński	28.0	1.45
	1421	Pruszkowski	34.9	1.54
	1422	Przasnyski	40.3	1.61
	1423	Przysuski	70.4	1.85
	1424	Pułtuski	56.4	1.75
	1425; 1463	Radomski aggregated	16.0	1.20
	1426; 1464	Siedlecki aggregated	276.2	2.44
	1427	Sierpecki	18.8	1.27
	1428	Sochaczewski	21.2	1.33
	1429	Sokołowski	79.1	1.90
	1430	Szydłowiecki	25.5	1.41
	1432; 1465	Warszawski aggregated	28.5	1.46
	1433	Węgrowski	133.5	2.13
	1434	Wołomiński	21.1	1.33
	1435	Wyszkowski	73.1	1.86

	1436	Zwoleński	54.4	1.74
	1437	Żuromiński	37.3	1.57
	1438	Żyrardowski	61.4	1.79
Opole	1601	Brzeski	34.2	1.53
	1602	Głubczycki	48.8	1.69
	1603	Kędzierzyńsko-Kozielski	65.3	1.81
	1604	Kluczborski	37.1	1.57
	1605	Krapkowicki	89.2	1.95
	1606	Namysłowski	34.4	1.54
	1607	Nyski	70.1	1.85
	1608	Oleski	28.7	1.46
	1609; 1661	Opolski aggregated	46.9	1.67
	1610	Prudnicki	31.9	1.50
	1611	Strzelecki	54.4	1.74
Subcarpathian	1801; 1808	Bieszczadzki & Leski aggregated	39.5	1.60
	1802	Brzozowski	86.5	1.94
	1803	Dębicki	55.4	1.74
	1804	Jarosławski	37.5	1.57
	1805	Jasielski	41.5	1.62
	1806	Kolbuszowski	155.4	2.19
	1807; 1861	Krośnieński aggregated	90.7	1.96
	1809	Leżajski	57.9	1.76
	1810	Lubaczowski	139.8	2.15
	1811	Łańcucki	53.4	1.73
	1812	Mielecki	14.8	1.17
	1813	Niżański	73.7	1.87
	1814; 1862	Przemyski aggregated	25.8	1.41
	1815	Przeworski	30.2	1.48
	1816	Ropczycko-Sedziszowski	27.7	1.44
	1817; 1863	Rzeszowski aggregated	34.3	1.54
	1818	Sanocki	34.3	1.54
	1819	Stalowowolski	26.7	1.43
	1820	Strzyżowski	24.8	1.40
	1821; 1864	Tarnobrzeski aggregated	40.5	1.61
Podlachian	2001	Augustowski	87.0	1.94
	2002; 2061	Białostocki aggregated	82.8	1.92
	2003	Bielski	163.5	2.21
	2004	Grajewski	144.1	2.16
	2005	Hajnowski	196.1	2.29
	2006	Kolneński	60.6	1.78
	2007; 2062	Łomżyński aggregated	36.0	1.56
	2008	Moniecki	50.1	1.70

	2009	Sejneński	213.3	2.33
	2010	Siemiatycki	103.5	2.02
	2011	Sokólski	169.7	2.23
	2012; 2063	Suwalski aggregated	134.5	2.13
	2013	Wysokomazowiecki	47.1	1.67
	2014	Zambrowski	29.6	1.47
Pomeranian	2201	Bytowski	142.9	2.15
	2202	Chojnicki	24.8	1.39
	2203	Człuchowski	22.0	1.34
	2204; 2261	Gdański aggregated	30.0	1.48
	2205	Kartuski	33.6	1.53
	2206	Kościerski	69.4	1.84
	2207	Kwidzyński	35.5	1.55
	2208	Lęborski	48.8	1.69
	2209	Malborski	10.2	1.01
	2210	Nowodworski	19.9	1.30
	2211	Pucki	37.0	1.57
	2212; 2263	Słupski aggregated	67.4	1.83
	2213	Starogardzki	22.8	1.36
	2214	Sztumski	25.7	1.41
	2215	Tczewski	15.6	1.19
	2216	Wejherowski	40.5	1.61
	2262	Gdyński	17.0	1.23
	2264	Spocki	39.8	1.60
Silesia	2402; 2461	Bielsko-Biała aggregated	47.5	1.68
	2413; 2462; 2471	Bytom & Piekary Śląskie & Tarnogórski aggregated	30.0	1.48
	2463; 2476	Chorzów & Świętochłowice aggregated	20.3	1.31
	2403	Cieszyn	101.1	2.00
	2404; 2464	Częstochowski aggregated	20.5	1.31
	2401; 2465	Dąbrowa Górnicza & Będzin aggregated	42.3	1.63
	2405; 2466; 2478	Gliwice & Zabrze aggregated	49.5	1.69
	2468	Jaworzno	120.1	2.08
	2469; 2470; 2474	Katowice & Mysłowice & Siemianowice Śląskie	50.3	1.70
	2406	Kłobuck	26.0	1.42
	2407	Lubliniec	42.3	1.63
	2409	Myszków	32.2	1.51
	2411	Racibórz	56.7	1.75
	2472	Ruda Śląska	28.5	1.46
	2412; 2473; 2479	Rybnicki & Żory aggregated	30.1	1.48
	2475	Sosnowiec	28.0	1.45

	2414; 2408; 2410; 2477	Tychy & Bieruńsko-Lędziński & Pszczyński & Mikołowski aggregated	23.7	1.37
	2415; 2467	Wodzisław Śląski & Jastrzębie aggregated	103.4	2.01
	2416	Zawiercie	47.2	1.67
	2417	Żywiec	96.9	1.99
Świętokrzyskie	2601	Buski	27.5	1.44
	2602	Jędrzejowski	3.9	0.60
	2603	Kazimierski	10.8	1.03
	2604; 2661	Kielecki aggregated	50.3	1.70
	2605	Konecki	11.2	1.05
	2606	Opatowski	28.2	1.45
	2607	Ostrowiecki	15.7	1.20
	2608	Pińczowski	20.3	1.31
	2609	Sandomierski	12.0	1.08
	2610	Skarżyski	53.8	1.73
	2611	Starachowicki	26.1	1.42
	2612	Staszowski	44.1	1.64
	2613	Włoszczowski	20.8	1.32
Warmian-Masurian	2801	Bartoszycki	51.8	1.71
	2802	Braniewski	95.3	1.98
	2803	Działdowski	33.8	1.53
	2804; 2861	Elbląski aggregated	17.5	1.24
	2805	Ełcki	85.1	1.93
	2806	Giżycki	143.3	2.16
	2818	Gołdapski	87.2	1.94
	2807	Iławski	41.7	1.62
	2808	Kętrzyński	44.9	1.65
	2809	Lidzbardzki	41.3	1.62
	2810	Mrągowski	147.3	2.17
	2811	Nidzicki	41.6	1.62
	2812	Nowomiejski	29.6	1.47
	2813	Olecki	86.4	1.94
	2814; 2862	Olsztyński aggregated	55.6	1.74
	2815	Ostródzki	141.4	2.15
	2816	Piski	98.3	1.99
	2817	Szczycieński	113.2	2.05
	2819	Węgorzewski	156.8	2.20
	3001	Chodzieski	28.0	1.45
	3002	Czarnkowsko-Trzcianecki	5.4	0.73
	3003	Gnieźnieński	8.0	0.91
	3004	Gostyński	2.8	0.44
	3005	Grodziski	11.7	1.07

	3006	Jarociński	10.5	1.02
	3007; 3061	Kaliski aggregated	8.8	0.94
	3008	Kępiński	15.1	1.18
	3009	Kolski	6.0	0.78
	3010; 3062	Koniński aggregated	7.0	0.84
	3011	Kościański	4.1	0.61
	3012	Krotoszyński	11.5	1.06
	3013; 3063	Leszczyński aggregated	8.8	0.94
	3014	Międzychodzki	12.2	1.09
	3015	Nowotomyski	21.9	1.34
	3016	Obornicki	6.7	0.83
	3017	Ostrowski	9.6	0.98
	3018	Ostrzeszowski	8.6	0.93
	3019	Pilski	26.5	1.42
	3020	Pleszewski	3.6	0.55
	3021; 3064	Poznański aggregated	17.0	1.23
	3022	Rawicki	9.1	0.96
	3023	Słupecki	6.9	0.84
	3024	Szamotulski	10.1	1.00
	3025	Śremski	11.7	1.07
	3026	Średzki	14.6	1.16
	3027	Turecki	6.1	0.79
	3028	Wągrowiecki	3.9	0.60
	3029	Wolsztyński	14.0	1.15
	3030	Wrzesiński	12.9	1.11
	3031	Złotowski	13.9	1.14
West Pomeranian	3201	Białogardzki	24.1	1.38
	3202	Choszczeński	20.2	1.30
	3203	Drawski	75.2	1.88
	3204	Goleniowski	21.1	1.32
	3205	Gryficki	61.0	1.79
	3206	Gryfiński	24.4	1.39
	3207	Kamieński	64.5	1.81
	3208	Kołobrzeski	32.5	1.51
	3209; 3261	Koszaliński aggregated	11.0	1.04
	3218	Łobeski	46.6	1.67
	3210	Myśliborski	29.9	1.48
	3211	Policki	34.7	1.54
	3212	Pyrzycki	13.6	1.13
	3213	Sławieński	40.7	1.61
	3214	Stargardzki	29.1	1.46
	3262	Szczeciński	29.4	1.47
	3215	Szczecinecki	24.6	1.39

	3216	Świdwiński	44.1	1.64
	3263	Świnoujście	88.0	1.94
	3217	Wałecki	58.6	1.77

Supplementary Table S2. Posterior distribution statistics from the most parsimonious model.

Variable	Mean	2.5%	97.5%
Forest cover (%)	0.0049	0.0025	0.0074
Public parks and private green areas (%)	0.0583	0.0327	0.0829
Minimum monthly temperature (ºC)	0.0425	0.0012	0.0800
Mean monthly precipitation (Log10 mm/month)	0.2730	-0.0282	0.5645
8-day gross primary productivity (gC/m^2)	0.0243	0.0117	0.0375
Human population density (Log10 ind/km2)	-0.2640	-0.3468	-0.1789
Nu2	0.0139	0.0114	0.0167
Tau2	0.1450	0.1304	0.1609
Rho.S	0.7447	0.6468	0.8353
Rho.T	0.8797	0.8505	0.9046

Supplementary Table S3. Potential covariates of Lyme borreliosis incidence

Category	Description (unit)	Rationale	Data source (reference)	Data resolution
Vegetation characteristics	Share of district area with forest cover (%)	Forests provide tick favorable habitat and increase chances of human-tick contact and sustain diverse ticks' hosts populations [6,7]	Local Data Bank [1]	District level
	Share of district area with forest cover dominated by deciduous tree species (%)	Deciduous forests and their permanent leaf litter layer generally provide suitable habitats and microclimate for ticks [6,8]	Forest Data Bank [2]	District level
	Share of district area with forest cover dominated by coniferous tree species (%)	Compared to deciduous forests, coniferous forests provide less suitable conditions for ticks in terms of humidity, frost susceptibility and temperature [6]	Forest Data Bank [2]	District level
	The share of district area covered by parks, lawns and green areas of the housing estates (%)	Green spaces increase both tick populations and the risk of tick-human contact, especially in parks and gardens [9,10]	Local Data Bank [1]	District level
	The density of large and small	Large and small wild mammals are suitable	Forest Data Bank [2]	District level

	wild mammal species (n/km^2)	hosts for ticks and essential to maintain *Ixodes* populations [14]		
Climate	Yearly minimum and maximum of monthly mean temperatures (^{o}C)	Ticks' host questing and tick development are influenced by temperature and its fluctuations by prolonging duration of ticks activity (also in winter) and increasing risk of tick-host contact and pathogen transmission [14-17]<br>Range of LB risk expands as increasing temperature shift local faunal abundance and diversity influencing ticks' host populations [14-18];<br>Ticks mortality increases if they are exposed to extreme or prolonged low temperatures [19]	TerraClimate [3]	~4 km, data aggregated to district level (using the mean)
	Yearly mean precipitation (mm/month) Log$_{10}$ transformed	Humidity is the principal determinant of inter-stadial mortality during the ticks development periods on the ground [13,20]	TerraClimate [3]	~4 km, data aggregated to district level (using the mean)
	Days with snow cover (n/year)	Snow cover may protect ticks in winter however, persistence of snow cover can delay the start of ticks' seasonal activity [17,19]	MODIS MOD10A1 [4,22]	500 m, data aggregated to district level (using the mean)
	Yearly mean 8-day gross primary productivity (GPP) (gC/m^2)	Plant productivity supports tick host abundance [23]	MODIS MOD17A2H [4,22]	500 m, data aggregated to district level (using the mean)
	Growing degree days (GDD) (^{o}C) Log$_{10}$ transformed	A longer growing season supports ticks' hosts populations and may give ticks more time to acquire the blood meal that enhances survivorship [24];<br>The timing of the activity peak and duration of the LB season are associated	ERA5 daily [5]	0.25 degree (~25 km), data aggregated to district level (using the mean)

		with cumulative growing degree days [25]		
Socio-economic	Number of medical staff (doctors and nurses) per 100,000 inhabitants Log_{10} transformed	May potentially be used to correct for observation bias as there is relationship between increasing number of LB cases and number of medical staff we can assume that disease detection is to low; A higher density of medical staff may increase LB diagnostics and health care access [26]; A higher number of tick-borne diseases-educated medical staff can increase prevention behaviors [27]	Local Data Bank [1]	District level
	Human population density (n/km^2) Log_{10} transformed	Transformation of natural environments into urban settlements negatively affect biodiversity and influence on tick-borne pathogens transmission [28], but increasing urbanization and the human behavior of encroaching on peri-urban surroundings rises the exposition of humans to vector ticks and tick-transmitted pathogens [9]	Local Data Bank [1]	District level

References of Supplementary Table S3

1. Statistics Poland – Local Data Bank; GUS - Bank Danych Lokalnych. n.d. https://bdl.stat.gov.pl/bdl/start.
2. The Forest Data Bank; Bank Danych o Lasach. n.d. https://www.bdl.lasy.gov.pl/portal/zestawienia-en.
3. Abatzoglou, J. T., Dobrowski, S. Z., Parks, S. A., Hegewisch, K. C. TerraClimate, a high-resolution global dataset of monthly climate and climatic water balance from 1958–2015. *Scientific Data.* **5,** 2018.
4. Hall, D.K., Riggs, G. A. MODIS/Terra Snow Cover Daily L3 Global 500m SIN Grid, Version 6. [Indicate subset used]. Boulder, Colorado USA. NASA National Snow and Ice Data Center Distributed Active Archive Center. https://doi.org/10.5067/MODIS/MOD10A1.006 (2016).
5. Copernicus Climate Data Store; n.d. https://cds.climate.copernicus.eu/cdsapp#!/home
6. Tack, W., Madder, M., Baeten, L., De Frenne, P., Verheyen, K. The abundance of *Ixodes ricinus* ticks depends on tree species composition and shrub cover. *Parasitology*. **139,** 1273–81 (2012).
7. Pfäffle, M. P., Littwin, N., Muders, S. V. & Petney, T. N. The ecology of tick-borne diseases. *Int. J. Parasitol.* **43,** 1059–1077 (2013).

8. Diuk-Wasser, M. A., VanAcker, M. C., Fernandez, M. P. Impact of land use changes and habitat fragmentation on the eco-epidemiology of tick-borne diseases. *J. Med. Entomol*. **58,** 1546–64 (2020). https://doi.org/10.1093/jme/tjaa209.
9. Oechslin, C. P. *et al.* Prevalence of tick-borne pathogens in questing *Ixodes ricinus* ticks in urban and suburban areas of Switzerland. *Parasit. Vectors.* **10,** (2017).
10. Heylen, D. *et al.* Ticks and tick-borne diseases in the city: Role of landscape connectivity and green space characteristics in a metropolitan area. *Sci. Total Environ.* **670,** 941–949 (2019).
11. Levi, T., Keesing, F., Holt, R. D., Barfield, M. & Ostfeld, R. S. Quantifying dilution and amplification in a community of hosts for tick-borne pathogens. *Ecol. App.* **26,** 484–498 (2016).
12. Brady, R. M., Lemieux, C. J., Doherty, S. T. Linking visitor perceptions and behaviours related to ticks and Lyme disease to risk management strategies in a protected areas context. *J. Outdoor Recreat. Tour.* **39,** 100515 (2022).
13. Randolph, S. E. Tick-borne disease systems. *Rev. Sci. Tech. Off. Int. Epiz.* **27,** 1-15 (2008).
14. Li, S., Gilbert, L., Harrison, P. A., Rounsevell, M. D. Modelling the seasonality of Lyme disease risk and the potential impacts of a warming climate within the heterogeneous landscapes of Scotland. *J. R. Soc. Interface.* **13,** 20160140 (2016).
15. Ogden, N. H. *et al.* Risk maps for range expansion of the Lyme disease vector, *Ixodes scapularis*, in Canada now and with climate change. *Int. J. Health Geogr*. **7,** 24 (2008).
16. Zając, Z. *et al.* Environmental determinants of the occurrence and activity of *Ixodes ricinus* ticks and the prevalence of tick-borne diseases in eastern Poland. *Sci. Rep.* **11**, (2021).
17. Dautel, H., Dippel, C., Kämmer, D., Werkhausen, A. & Kahl, O. Winter activity of Ixodes ricinus in a Berlin forest. *Int. J. Med. Microbiol.* **298,** 50–54 (2008).
18. McPherson, M., *et al.* Expansion of the lyme disease vector *Ixodes scapularis* in Canada inferred from CMIP5 Climate Projections. *Environ. Health Perspect*. **125,** (2017).
19. Dautel, H. & Knülle, W. Cold hardiness, supercooling ability and causes of low-temperature mortality in the soft tick, *Argas reflexus*, and the hard tick, *Ixodes ricinus* (Acari: Ixodidae) from Central Europe. *J. Insect Physiol.* **43,** 843–854 (1997).
20. Cunze, S., Glock, G., Kochmann, J. & Klimpel, S. Ticks on the move—climate change-induced range shifts of three tick species in Europe: current and future habitat suitability for *Ixodes ricinus* in comparison with *Dermacentor reticulatus* and *Dermacentor marginatus*. *Parasitol. Res.* **121,** 2241–2252 (2022).
21. Randolph, S. & Storey, K. M. Impact of microclimate on immature tick-rodent host interactions (Acari: Ixodidae): Implications for parasite transmission. *J. Med. Entom.* **36,** 741–748 (1999).
22. Running, S., Mu, Q., Zhao, M. MOD17A2H MODIS/terra gross primary productivity 8-day L4 global 500m SIN grid V006. NASA EOSDIS Land Processes DAAC. 2015.
23. Cumming, G. S. Comparing climate and vegetation as limiting factors for species ranges of African ticks. *Ecology*. **83,** 255–68 (2002).
24. Kaizer, A. M., Foré, S. A., Kim, H. J., York, E. C. Modeling the biotic and abiotic factors that describe the number of active off-host *Amblyomma americanum* larvae. *J. Vector Ecol.* **40,** 1–10 (2015).
25. Moore, S. M., Eisen, R. J., Monaghan, A., Mead, P. Meteorological influences on the seasonality of Lyme disease in the United States. *Am. J. Trop. Med. Hyg.* **90,** 486–96 (2014);
26. Johnson, L., Aylward, A., Stricker, R. B. Healthcare access and burden of care for patients with Lyme disease: A large United States survey. *Health Policy*. **102,** 64–71 (2011);
27. Howard, K. *et al.* Assessment of knowledge, attitudes, and practices toward ticks and tickborne disease among healthcare professionals working in schools in New York and Maryland. *J. Sch. Nurs.* 105984052210994 (2022).
28. Léger, E., Vourc'h, G., Vial, L., Chevillon, C., McCoy, K. D. Changing distributions of ticks: causes and consequences. *Exp. App. Acarol*. **59,** 219–44 (2012).

Supplementary Figure S1. Mean Lyme borreliosis incidence per 100,000 inhabitants ($\log_{10}$ transformed) from 2010 to 2019 for 320 aggregated districts. Territorial identifiers correspond to Supplementary Table S1. This map was generated based on the compiled LB incidence data with the use of an online tool (https://www.datawrapper.de/).

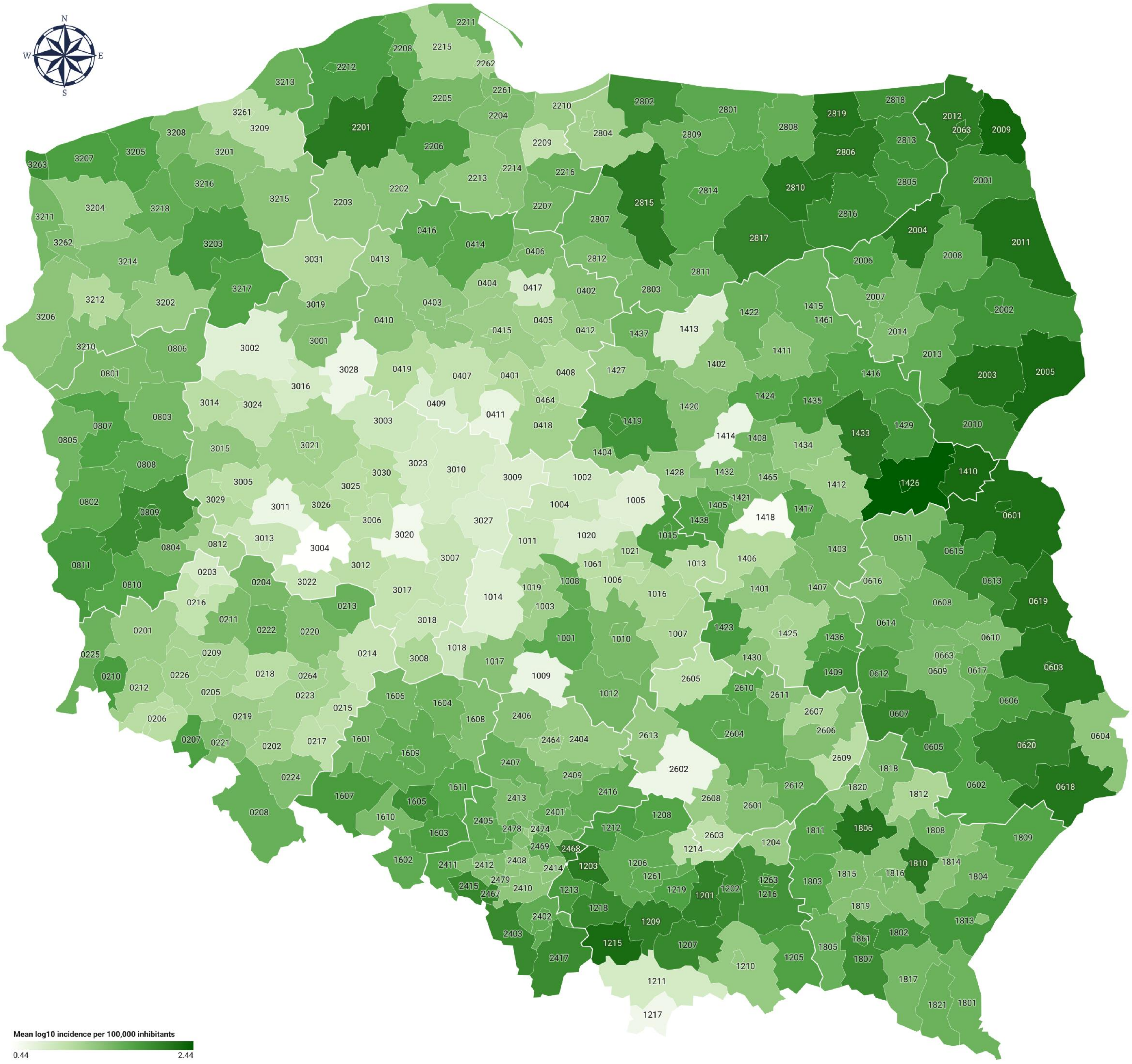

Supplementary Figure S2. Observed and predicted time trends of LB incidence. Blue dots represent the yearly specific observed LB incidence calculated as the median over all Polish districts, red squares depict the median predicted LB incidence for all districts. This figure was generated with use of R (version 4.3.2) using ggplot2 (version 3.4.4).

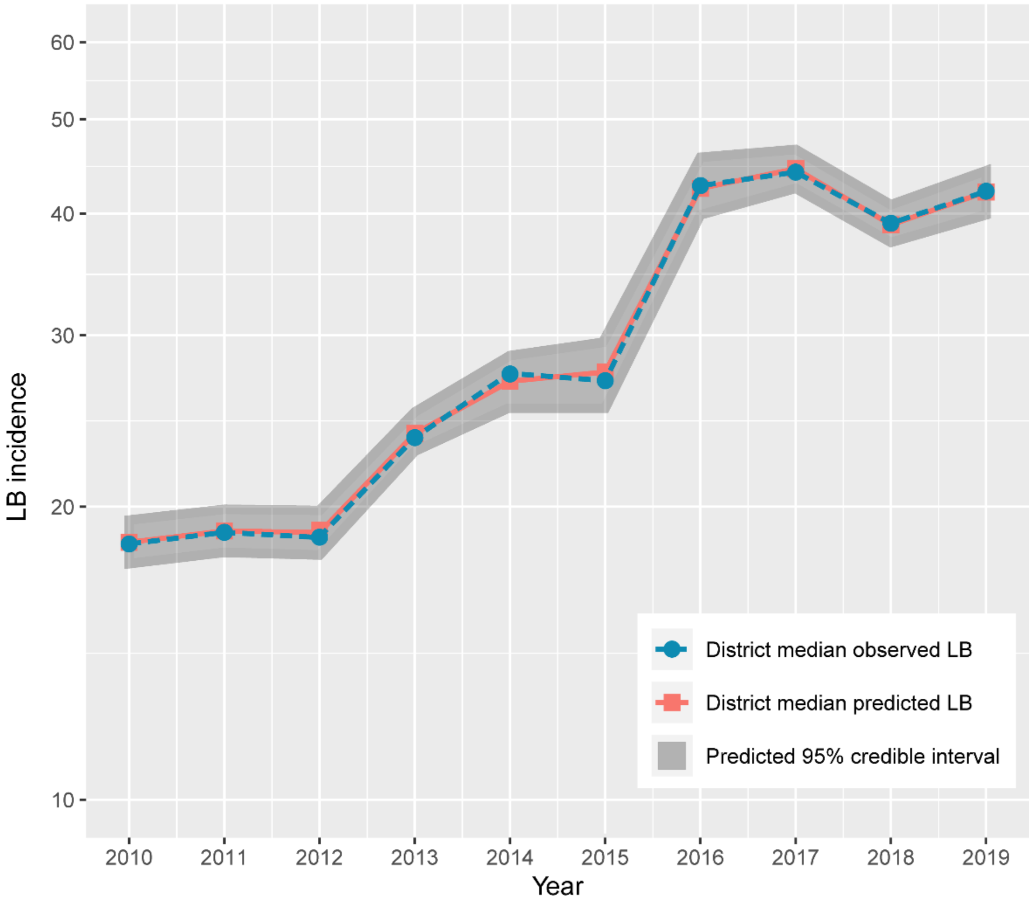

Supplementary Figure S3. Histograms of the covariates and LB incidence used for model selection.

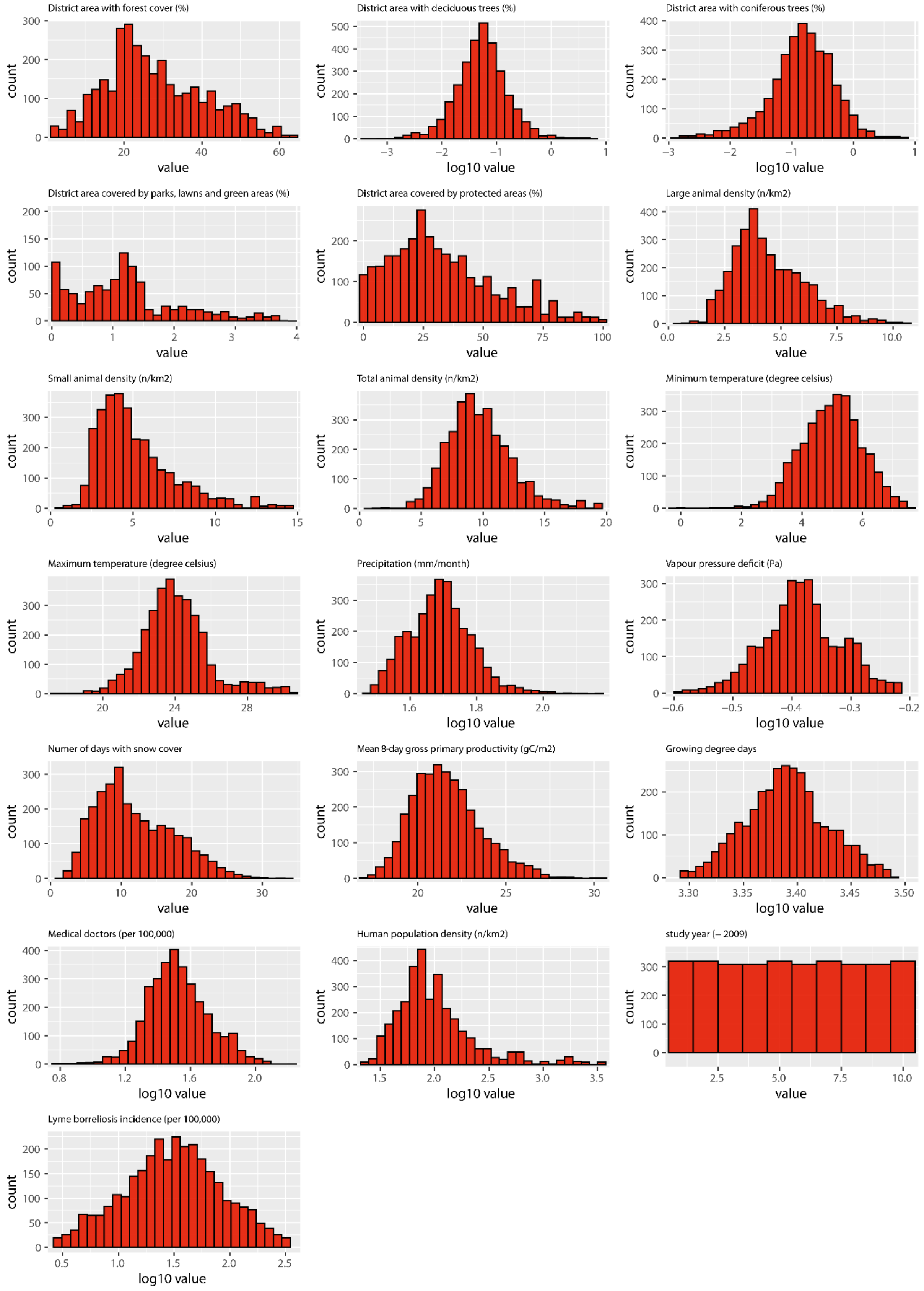

Supplementary Figure S4. The trace and density plots of the MCMC samples from 3 chains for the intercept and all variables included in the final model.

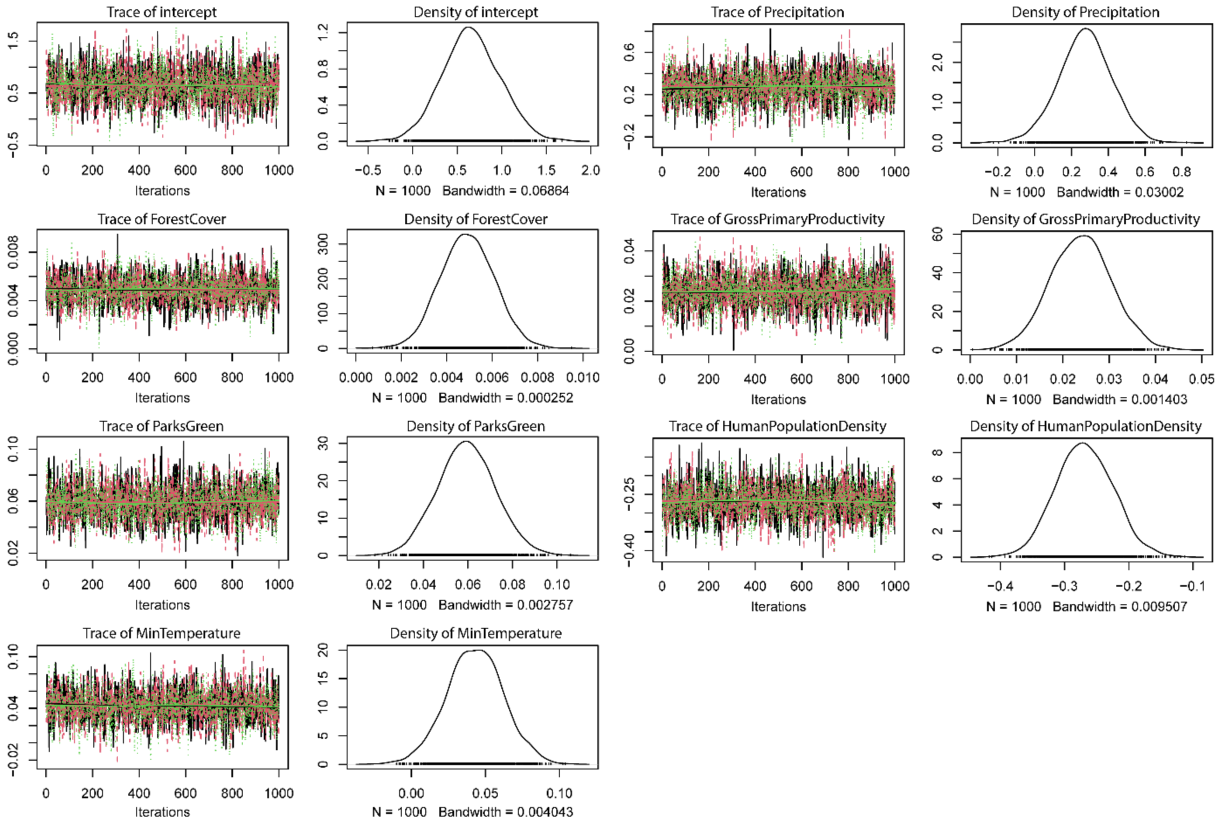

Supplement: Supplementary file 1 — Supplementary Information. [file 41598_2024_61349_MOESM1_ESM.pdf]
